# Supplementary material for: Flotillin-1 promotes EMT of gastric cancer via stabilizing Snail
Source: PeerJ. 2022 Aug 15;10:e13901. doi: 10.7717/peerj.13901 (PMC9387518; doi:10.7717/peerj.13901)
Supplement: Supplemental Information 3 [file peerj-10-13901-s003.docx]

**Supplementary Tables**

**Table S1 The sequences of shRNAs**

| Sequence (5’ to 3’) | Name |
| --- | --- |
| GGAAGTACTGGACATTCTAAC | shFlotillin-1-1 |
| CCCTCAATGTCAAGAGTGAAA | shFlotillin-1-2 |

**Table S2 The primers used in qRT-PCR**

| Sequence (5’ to 3’) | Name |
| --- | --- |
| TTGCACCGGTCGACAAAGGAC | E-cadherin Forward |
| TGGATTCCAGAAACGGAGGCC | E-cadherin Reverse |
| TGTCGGTGACAAAGCCCCTG  AGGGCATTGGGATCGTCAGC  ACCCGCACCAACGAGAAGGT  ATTCTGCTGCTCCAGGAAGCG  CTGGGTGCCCTCAAGATGCA | N-cadherin Forward  N-cadherin Reverse  Vimentin Forward  Vimentin Reverse  Snail Forward |
| CCGGACATGGCCTTGTAGCA | Snail Reverse |
